# Effectiveness of Family-Involved Interventions in Reducing Co-Occurring Alcohol Use and Mental Health Problems in Young People Aged 12–17: A Systematic Review and Meta-Analysis

**DOI:** 10.3390/ijerph20196890

**Published:** 2023-10-06

**Authors:** Emma Geijer-Simpson, Eileen Kaner, Raghu Lingam, Paul McArdle, Ruth McGovern

**Affiliations:** 1 Population Health Sciences Institute, Newcastle University, Newcastle upon Tyne NE2 4AX, UK; 2 School of Women’s and Children’s Health, Faculty of Medicine, University of New South Wales, Sydney, NSW 2031, Australia; 3 Northumberland, Tyne & Wear Foundation Trust, Newcastle upon Tyne NE3 3XT, UK

**Keywords:** young person, family-involved intervention, co-occurring, alcohol use, mental health problems, systematic review

## Abstract

There is a high prevalence rate of co-occurring alcohol use and mental health problems in young people. This is associated with adverse outcomes and poses a substantial public health concern. We identified and synthesized evidence on the effectiveness of family-involved interventions in reducing alcohol use and mental health problems in young people aged 12–17. Seven databases were searched from inception to January 2023. Data from 19 articles reporting on 14 trials were pooled through random-effects meta-analysis for each outcome using Review Manager 5.3. Pooled estimates resulted in non-significant findings for alcohol use (SMD −0.60; 95% CI −1.63 to 0.42; *p* = 0.25; 6 trials; 537 participants), internalizing symptoms (SMD −0.13; 95% CI −0.37 to 0.10; *p* = 0.27), externalizing symptoms (SMD −0.26; 95% CI −0.66 to 0.15; *p* = 0.22) and substance use (SMD −0.33; 95% CI −0.72 to 0.06; *p* = 0.10). In contrast, significant intervention effects were identified for the mechanism of change, family conflict (SMD −0.30; 95% CI −0.51 to −0.09; *p* = 0.005). Consequently, addressing family functioning may not be sufficient in reducing co-occurring alcohol use and mental health problems. Non-significant intervention effects could be due to a lack of content addressing the relationship between alcohol use and mental health problems. Future intervention development could explore whether to incorporate such content and how best to involve the family.

## 1. Introduction

Mental health and substance use disorders are the 6th leading contributors to the global burden of disease in young people below the age of 24 [1], measured as disability-adjusted life years (DALYs). Alcohol is the most widely used psychoactive substance in adolescent populations [2]. Here, alcohol use will be used to refer to any use, experimentation and irregular to frequent heavy use, which may reach clinical levels for abuse or dependence [3,4]. The co-occurrence of alcohol use and mental health problems are associated with poor school performance and dropout [5,6], legal problems [5], suicidal ideation [7,8], poorer treatment outcomes [9] and poorer health outcomes compared to those young people with either alcohol use or mental health problems separately [10,11]. Further co-occurring alcohol use and mental health problems in young people can lead to longitudinal effects into adulthood [12]. Consequently, adolescence provides an optimal opportunity to intervene with the potential to impact the entire lifespan [13].

Mental health problems, including internalizing problems (emotional problems) and externalizing problems (behavioral problems), frequently co-occur with alcohol use in young people [14,15,16,17]. A systematic review reported that up to 60% of young people aged 14 to 18 years who engage in alcohol and other substances also have co-occurring mental health problems (internalizing or externalizing problems) [6]. More recently, an England-based survey in 2017 found that rates of alcohol use and frequency of alcohol use were higher in those young people with clinical levels of mental health problems compared to those without [17]. Specifically, 36% of young people aged 11–16 with a formally diagnosed mental disorder had tried alcohol, compared to 22.7% without a mental health disorder [17]. Similarly, 31.7% of young people with a mental health disorder were more likely to drink monthly, in contrast to 19.4% of those without a mental disorder [17]. However, these estimates may be an underrepresentation as many people experience co-occurring problems without meeting the threshold for a diagnosis by a health and care professional [18]. With mental health and alcohol use problems presenting on a continuum, sub-threshold levels can still lead to detrimental outcomes [19]. Examining sub-threshold levels, Lewinsohn and colleagues reported that 33% of young people with conduct problems and 27.8% of young people with depression had co-occurring alcohol problems [18]. Studies such as Lewinsohn et al., which report subthreshold prevalence estimates for young people, are few [20].

**Figure 1:**
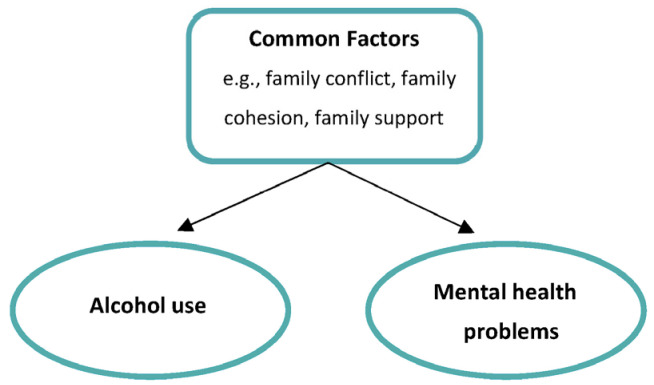
Common factor model.

One theoretical model delineating the possible cause of co-occurring mental health problems and alcohol use is the common factor model. This model suggests that risk and protective factors are not problem/disorder specific but rather that alcohol and mental health problems may be a result of common underlying factors, including factors relating to the family [13]. Please see Figure 1. Whilst studies suggest that heritability plays an important role in co-occurring alcohol use and mental health problems [21,22], external factors have also been found to be key. Studies have identified protective factors, including emotional closeness, bonding with family, carers rewarding good behavior and family cohesion [23], opportunities and rewards for prosocial involvement and attachment to be associated with subthreshold and clinical levels of co-occurring alcohol use and mental health problems. They have also identified a range of familial risk factors to be associated with co-occurring alcohol use and mental health problems. These include family conflict, family history of antisocial behavior and substance use [20,24], parental attitudes favorable toward drug use and antisocial behavior [24], poor family management, and low levels of familial support [25]. Targeting these risk and/or protective factors offers an opportunity for the prevention and early treatment of a broad range of outcomes, including both alcohol use and internalizing and externalizing symptoms [13]. Increasingly, the need for the prevention of alcohol use and mental health problems, alongside treatment, is recognized [13,16,26]. Prevention aims to delay the onset or initiation and reduce levels of symptoms before they reach a diagnostic threshold [27]. As such, this review encompasses both.

There is a dearth of psychosocial interventions that have been specifically developed for co-occurring alcohol use and mental health problems in young people [14,28]. With the frequent co-occurrence of alcohol use and mental health problems and shared risk/protective factors, this could be an efficient and effective approach. Family-involved interventions are designed to target these shared underlying familial factors, therefore having the potential to address co-occurring alcohol use and mental health problems [29]. For many family-involved interventions, the primary mechanism of change is often indirect. Here, emotions, cognitions and behaviors within the family are targeted to improve family functioning. The improved family functioning is, in turn, theorized to reduce the risk of a range of outcomes, including alcohol use and mental health problems [30]. There are also interventions that may not employ family functioning as the mechanism of change; however, they include family involvement. For example, the National Institute of Health and Care Excellence (NICE) recommends including family members within interventions for young people ranging from 10–17 to prevent clinical levels of alcohol use [31]. Family-involved interventions have been found to be effective in reducing both subthreshold and clinical levels of mental health problems, alcohol use and wider substance use separately [32,33,34,35]. However, less is known about the effectiveness of family-involved interventions for co-occurring alcohol use and mental health problems (internalizing and externalizing problems).

We conducted a systematic review and meta-analysis of randomized controlled trials and quasi-experimental trials to identify and assess the effectiveness of family-involved interventions in preventing and reducing co-occurring alcohol use and mental health problems (internalizing and externalizing symptoms) in young people aged 12–17.

## 2. Materials and Methods

The review protocol was preregistered on Prospero—CRD42016039147

### 2.1. Eligibility Criteria

Studies were deemed eligible for inclusion if they:

1. Targeted young people aged 12–17 of any ethnicity or gender. Twelve years was selected as the lower cut-off as it is the common age of onset for both alcohol use and mental health problems [36,37]. Seventeen was selected as the upper age limit as family does not always remain a key influential context beyond this age. This is partly due to no longer having legislated age-related restrictions in the UK and other European countries [38]. Therefore, alcohol use may be less dependent on the family’s influence. Trials that had a broader age range were included if the mean age of participants fell between 12–17 years.
2. Reported on a family-involved psycho-social intervention in which a young person and caregiver were included, either separately or together, in at least one session. A broad definition of family was employed to include parents, carers, grandparents, aunts, uncles and siblings. All levels of prevention and treatment were included to ensure a more thorough evaluation and to enable comparisons between these three levels of family-involved psycho-social interventions. These levels include: ‘universal prevention’ targets the entire population irrespective of risk [39]; ‘targeted prevention’ consists of ‘selective’ interventions [39]; targeting individuals at risk and ‘indicated’ interventions [39]; individuals with pre-existing symptoms or pre-clinical diagnoses, with the aim of reducing alcohol use and mental health problems before it reaches a diagnostic threshold [39] and ‘treatment’ is aimed at individuals with a diagnosis [13]. Levels of prevention can be considered to be on a continuum, with the levels merging into one another rather than occurring as distinct alternatives [40].
3. Reported on both the primary outcomes: alcohol consumption (including frequency of drinking, binge drinking defined as drinking five or more drinks on any one occasion, regular or problem drinking) and common adolescent mental health problems including (a) internalizing problems such as anxiety and depression as well as (b) externalizing problems such as conduct problems and ADHD symptoms. Outcome measures could either report on the specific mental health problems or the overall internalizing or externalizing symptom score. Secondary outcomes included other substances and family functioning.
4. Had a robust evaluation design, specifically randomized controlled trials (RCTs), controlled trials, randomized trials (RTs) and quasi-experimental trials. Trials that included active controls (such as a different variant of the same intervention or a different kind of therapy) were defined as RTs, and those employing inactive controls (such as no treatment, waitlist control and standard care) were defined as RCTs in this review [41].

Trials were excluded if they were limited to young people with experience of trauma, such as sexual assault, domestic violence and abuse; or specific care needs, e.g., autistic spectrum disorder, learning difficulties or cancer; or with unique environmental circumstances, including refugee, war-torn/disaster zone, military families and homelessness. Furthermore, trials were excluded if they did not report on a measure of alcohol use either separately or within a composite measure (alcohol and other drugs together).

### 2.2. Search Strategy

The following databases were searched from inception to January 2023 without language, year or publication status restrictions: MEDLINE (OVID), PsycINFO (OVID), Web of Science (EBSCO), The Cochrane Central Register of Controlled Trials (OVID), CINAHL (EBSCO), ASSIA (Proquest) and Embase (OVID). The search strategy included a combination of medical subject headings/thesaurus headings, appropriate keywords and free text terms applying boolean, proximity and truncation operators. This approach was supplemented with a search of the grey literature and relevant journals, e.g., *Journal of Adolescent Health*, *Journal of Youth and Adolescence* and *Journal of Child and Family Studies*. Here, combinations of the keywords developed in the search strategy were used. Citations and references of included trials were also screened. The search strategy is available as a Supplementary File.

### 2.3. Study Selection, Risk of Bias Assessment and Extraction

**Figure 2:**
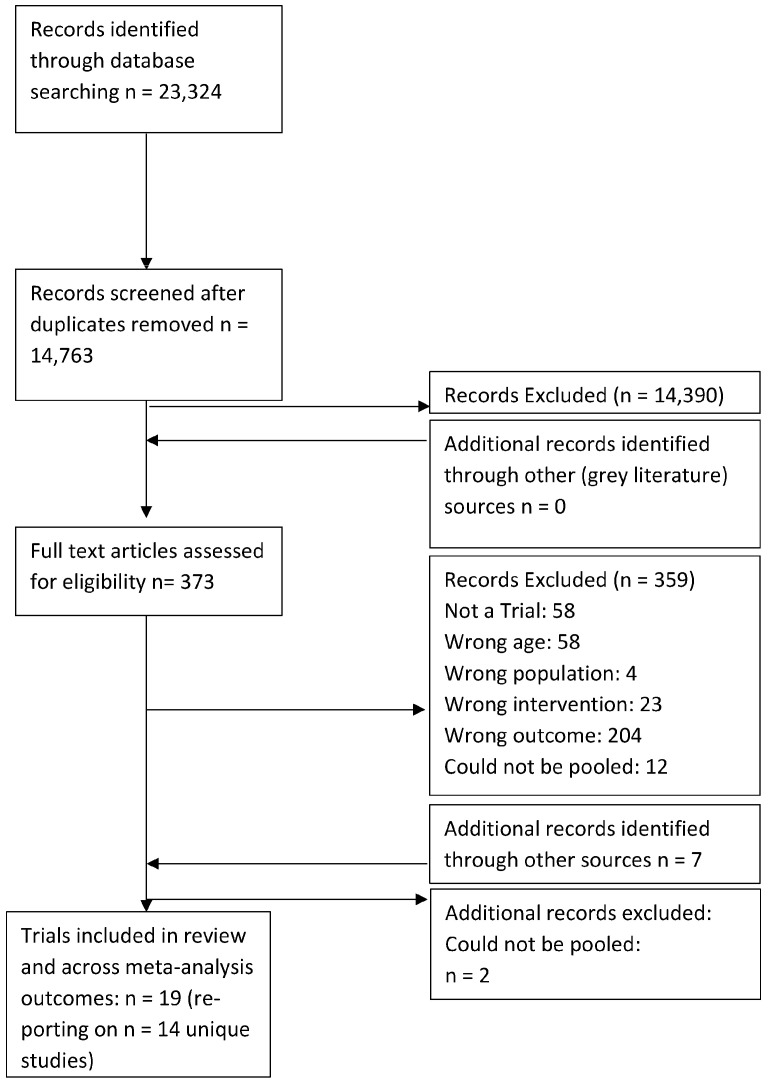
Prisma flow diagram.

Two researchers independently screened all titles and abstracts, followed by full-text review of eligible trials against pre-specified inclusion/exclusion criteria. Please see Figure 2. Two researchers also data-extracted and appraised the methodological quality of the trials using the Cochrane Collaboration’s risk of bias tool, which assesses selection, performance, detection, attrition and reporting bias [42]. Trials were not excluded based on the quality appraisal; rather, it informed critical evaluations of the conclusions of included trials. A third researcher resolved disagreements arising at any stage.

### 2.4. Data Analysis

We conducted a random-effects meta-analysis for each outcome using Review Manager 5.3. This method was employed due to perceived levels of heterogeneity and to enable the ability to generalize findings beyond the analytic sample. Continuous data were analyzed using weighted standardized mean differences between the family-involved interventions and control groups to produce a pooled effect size and 95% confidence intervals (Cis). Due to the small number of studies within prevention and treatment, they were pooled together, followed by sub-group analysis. Arguably, categories of prevention and treatment can be seen as on a continuum, with the levels merging into each other rather than distinct alternatives and were therefore able to be pooled together [40]. Other pre-planned subgroup analysis included age and duration of intervention. Age was explored in relation to effectiveness as 12–17 is a broad age range, and whilst family remains an influential context, young people progressively seek autonomy and peers become an increasingly influential source [43]. It is acknowledged that subgroup analysis can be underpowered, which can lead to Type II errors. However, it is still deemed important to carry out whilst interpreting findings with caution. Further, to minimize the risk of type I error, only a small set of pre-specified subgroup analyses should be carried out, as performed within this review [44]. Where possible, intention to treat data was used to examine group differences at longest follow-up time point for the primary outcome measures: (1) frequency of alcohol use (number of days of alcohol use in the past month), (2a) mental health: externalizing symptoms, (2b) mental health: internalizing symptoms and secondary outcome measures: (3) family conflict and (4) frequency of substance use (number of days of substance use in the past month). Family conflict was included to explore the mechanism of change. To maximize the number of studies that could be pooled, the longest follow-up time point was used. It was not possible to use intervention duration to inform this decision as this was rarely reported. However, the impact of applying the longest follow-up time point was explored within a sensitivity analysis outlined below. Youth self-reporting was prioritized over caregiver or teacher reporting. If means and standard deviations were not provided, authors were contacted and provided data was used in the analysis [45,46]. Where standard errors were reported, these were converted into standard deviations [47]. If trials had more than one intervention or control group, then these were pooled [48,49]. Levels of heterogeneity and statistical significance were assessed based on the I^2^ value and Chi^2^ test, respectively, and a *p* value of *0.10* was applied as outlined in the *Cochrane Handbook* [50]. In keeping with Cochrane guidance, the following cut-offs were applied: 0–40%, which might not be important; 30–60% with moderate heterogeneity; 50% to 90% with substantial heterogeneity and 75% to 100% with considerable heterogeneity [50].

Sensitivity analysis was conducted to investigate the effect of omitting trials that did not report follow-up time points falling within a time band of 3–12 months. This time band was applied as three months was the modal time point, and 12 months was the shortest follow-up time point for one of the trials. This analysis examined the impact of applying the longest follow-up time point and the heterogeneity introduced by variation in follow-up time points ranging from post-test to five years post-baseline. Further sensitivity analyses were applied, omitting trials that did not report on a composite measure of substance use (most frequently reported outcome) and trials not reporting on overall internalizing or externalizing symptom score (most frequently reported outcome). Finally, a sensitivity analysis removing outliers was applied.

Most meta-analyses pooled less than 10 trials. As such, it was not possible to assess potential publication bias. There were 10 trials within the externalizing meta-analysis; however, the sample sizes of the included trials were similar, and therefore, according to the *Cochrane Handbook*, it was not deemed suitable to test for publication bias.

## 3. Results

### 3.1. Description of Included Trials

After deduplication, the search identified 14,763 articles. After title and abstract screening, 14,390 were excluded and an additional 359 articles were removed after full paper screening. Five trials were identified through additional sources. Specifically, the reference list of included studies. These five papers were linked to three of the included trials. This resulted in the inclusion of 19 articles reporting on 14 unique trials [45,46,47,48,49,51,52,53,54,55,56,57,58,59,60,61,62,63,64]. Please see Figure 2.

Eight trials were randomized controlled trials (RCTs) [45,46,48,49,51,56,57,61]. Six trials were randomized trials which evaluated two or more active interventions [47,54,55,58,59,64]. All trials were conducted in the USA, with the exception of one trial conducted in Australia [47]. The 14 trials involved 1840 young people (and families) with a mean age of 15.23 years (SD = 0.69) and an average percentage of females being 43.07 (SD = 15.49). Three of the 14 trials limited recruitment to specific ethnic groups, specifically Hispanic young people [51,54,64]. One study did not report on ethnicity [47]. Three studies had a more even split of Caucasian and minority/multiracial families [46,57,59], three a majority of Caucasian families [46,55,61] and the remaining trials included a majority of multiracial or minority families. The trials varied considerably in the familial socio-demographics reported. As such, they cannot be synthesized. Please see Table 1 for a summary of trial characteristics.

None of the trials examined the effectiveness of universal interventions. Four trials examined targeted interventions [46,51,54,64]. Ten trials evaluated treatment [45,47,48,49,55,56,57,58,59,61]. One of these trials reported on alternate treatment-based intervention focused on young people but with additional caregiver involvement (as opposed to the interventions primarily targeting family functioning). Only three out of the 14 interventions were specifically aimed at preventing and reducing internalizing mental health problems rather than simply including an internalizing outcome measure [46,47,51].

All interventions included content on family functioning and/or parent training. Family functioning components included strengthening the co-parenting alliance, joint problem solving, communication skills, reducing family conflict and behavioral contracting. Parent training included caregiving practices involving monitoring and setting limits, establishing clear norms and expectations and self-care. Five trials explicitly outlined, albeit to varying degrees, the addition of components delivered to the young person separately, targeting factors beyond family functioning [46,54,58,59,61]. These components included self-regulation, cognitive appraisal, goal setting, coping efficacy and strategies, problem-solving, motivation to change, alcohol and wider substance use refusal skills [46,58,59,61], and the relationship between alcohol (and wider substance use) depression [46] and distress [59]. Some also included components addressing external factors such as peers [46,54] and school, as well as racial-, cultural- and community-related issues [54,58].

Eight of the interventions included separate sessions for young people and caregivers alongside whole family sessions [45,46,49,51,55,61,64,65]. Two [47,51] ran separate caregiver sessions combined with whole family sessions. Two [45,64] involved whole family sessions only, and one did not involve any whole family sessions [55]. One intervention did not specify the nature of family involvement [57]. Mothers were the main family members involved in interventions. Further, only three interventions involved family members beyond primary caregivers [46,47,51].

Among these interventions, two were group-based and delivered with other families/caregivers/young people [47,51]. One trial included both caregivers where possible [55]. Three trials included other family members beyond caregivers [46,47,64].

Interventions were based on a variety of theoretical approaches. The majority applied family systems theory [45,47,48,49,54,58,59,64]. Three drew upon ecological systems theory with a specific focus on the family system [46,51,57]. The remaining two included social cognitive learning theory [61] and developmental psychopathology [46].

Control groups within RCTs included waitlist control [46,56] and standard care [45,48,49,51,57,61]. Randomized trials evaluated two or more active interventions [47,54,55,58,59,64], usually alternate therapy [47,54,55,58,59,64]. For five of these six trials, the alternate therapy consisted of a limited form of family involvement [47,54,55,58,59]. Two trials included more than one control [48,59].

### 3.2. Risk of Bias

**Figure 3:**
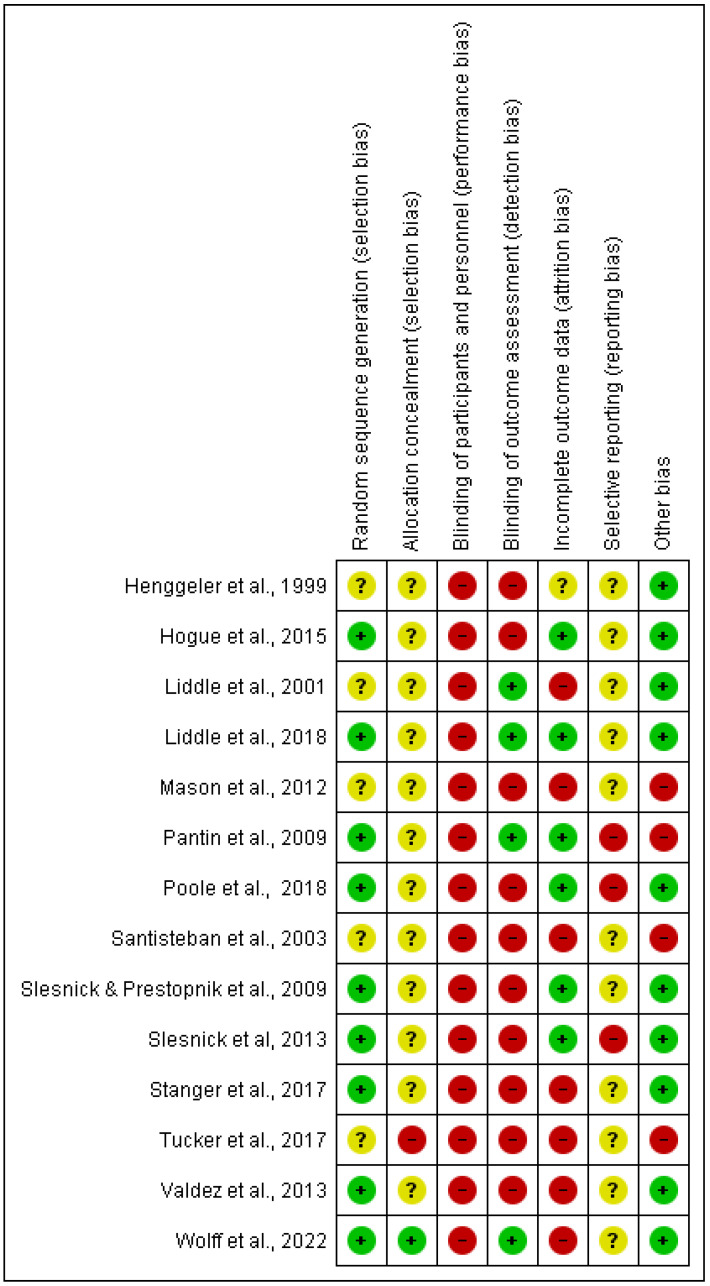
Risk of bias summary table.

Outlined below are the risk of bias appraisals for each of the 14 unique trials. Please see Figure 3 for a summary (green represents low risk, amber unclear risk and red high risk of bias).

#### 3.2.1. Random Sequence Generation

Nine studies were judged as having a low risk of bias [45,47,48,49,51,54,55,58,61]. These studies utilized a computer-generated random number sequence [54], urn randomization [45,48,49,51,58,61], block randomization procedure [47] and minimum likelihood allocation [55]. The remaining five trials were not clear about the method of sequence generation [46,56,57,59,64].

#### 3.2.2. Allocation Concealment

One trial provided sufficient detail to establish that participant allocation to experimental groups was concealed from those conducting the research; therefore, this trial was rated low risk of selection bias for this domain [61]. One study was considered to be at high risk [56] in which randomization occurred before enrolment. It was not possible to make a clear judgement regarding allocation concealment for the remaining 12 trials and were labelled as unclear [45,46,47,48,49,51,54,55,57,58,59,64].

#### 3.2.3. Blinding of Participants and Outcome Assessment

In all studies, blinding of participants and program deliverers (performance bias) was not achievable due to the nature of the interventions tested and because the outcomes were self-reported; therefore, we rated these studies as having a high risk of performance bias. In four studies, blinding of outcome assessment (detection bias) was rated as low risk as they explicitly stated efforts to blind assessors to group assignment upon outcome assessments [51,58,59,61].

#### 3.2.4. Incomplete Outcome Data

Six trials [45,47,48,49,51,58] were found to have a low risk of bias for incomplete outcome data, as they reported less than 20% loss of participants and of which five also showed no differential attrition between experimental groups [45,48,49,51,58]. Whilst one addressed missing data using statistical procedures [47] and was, therefore, also rated as low risk. Seven studies had a high risk of bias due to high attrition rates (>20%) or had less than 20% loss of participants but unequal attrition between experiment groups [46,54,55,56,59,61,64]. One study was rated as having unclear risk for incomplete outcome data, as details were insufficient to permit a judgement [57].

#### 3.2.5. Selective Reporting

Two studies were deemed at high risk [47,51]. One of these studies was deemed high risk due to not reporting three [47] outcomes outlined in the study protocol. The other was judged to be at high risk due to not providing a direct comparison for the experimental and control groups [51]. It was not possible to make a clear judgment regarding selective reporting for the remaining 12 studies.

#### 3.2.6. Other Potential Sources of Bias

We assessed ten trials as low risk to other forms of potential bias [45,47,48,49,54,55,57,58,59,61]. We judged four studies to be at high risk [46,51,56,64]: one due to lack of follow-up assessments [64], one due to lack of reporting results for the control group and incorrect labelling of follow-up time points [46] and two due to offering additional services and interventions alongside the intervention and/or control being assessed [51,56].

### 3.3. Meta-Analyses Findings

The meta-analysis findings are outlined for both the primary and secondary outcomes. Where sufficient numbers of trials were available to carry out sub-group analysis, this will be presented.

#### 3.3.1. Primary Outcomes

(1 Alcohol use: Frequency of use in the past 30 days (*n* = 6 trials; 3 targeted and 3 treatment)

##### Effectiveness of Family-Involved Interventions

**Figure 4:**
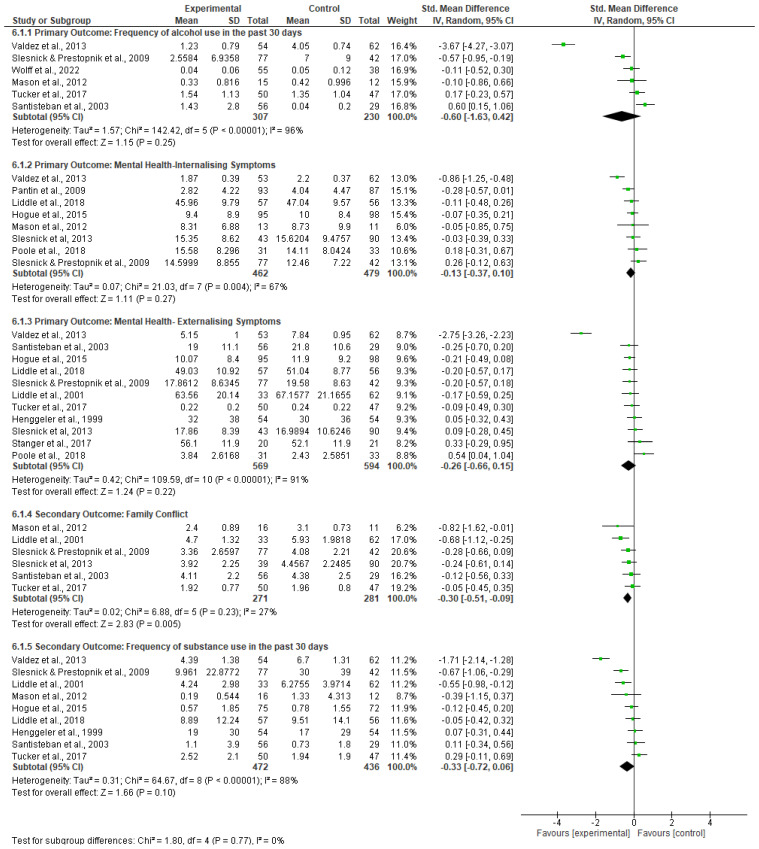
Impact of family-involved interventions on primary and secondary outcomes.

There was no significant difference between the family-involved interventions and the control groups at the longest follow-up time point (SMD −0.60; 95% CI −1.63 to 0.42; *p* = 0.25; 6 trials; 537 participants). There was considerable and significant heterogeneity between studies (I^2^ = 96%, *p* < 0.10)—(see Figure 4).

##### Impact of Prevention and Treatment

**Figure 5:**
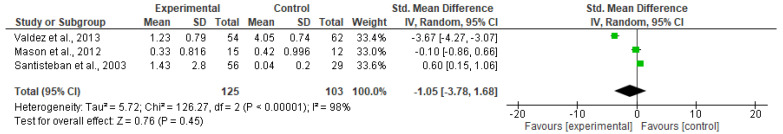
Targeted: frequency of alcohol use.

**Figure 6:**
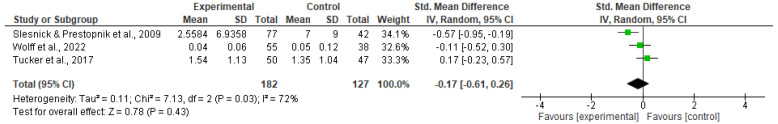
Treatment: frequency of alcohol use.

The effects of the intervention on the frequency of alcohol use were examined by level of prevention and treatment, analyzing separately targeted interventions and treatment-based interventions. Results remained non-significant; neither targeted nor treatment-based family-involved interventions reduced the frequency of alcohol use. See Figure 5 and Figure 6.

(2a) Mental Health: Internalizing Symptoms *(n* = 8 trials; 3 targeted and 5 treatment)

##### Effectiveness of Family-Involved Interventions

No significant difference was found between family-involved interventions and the control groups at the longest follow-up time point (SMD −0.13; 95% CI −0.37 to 0.10; *p* = 0.27; 8 trials; 941 participants). Heterogeneity levels demonstrated substantial and significant heterogeneity (I^2^ = 67% *p* < 0.10)—(see Figure 4).

##### Impact of Prevention and Treatment

**Figure 7:**
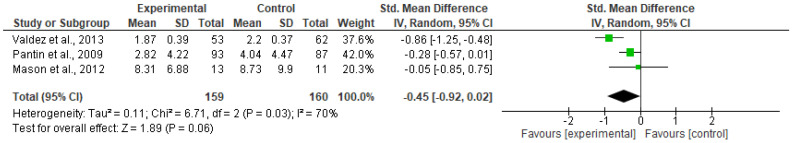
Targeted: Internalizing symptoms.

**Figure 8:**
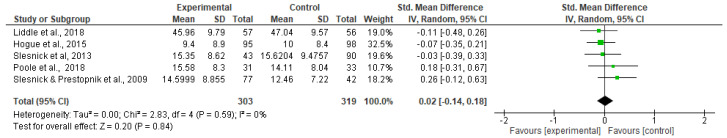
Treatment: Internalizing symptoms.

The effects of the intervention upon internalizing symptoms were examined by level of prevention and treatment, analyzing targeted interventions and treatment separately. Results remained non-significant; neither targeted nor treatment-based family-involved interventions reduced internalizing symptoms. Please see Figure 7 and Figure 8.

##### Impact of Young Person’s Age

**Figure 9:**
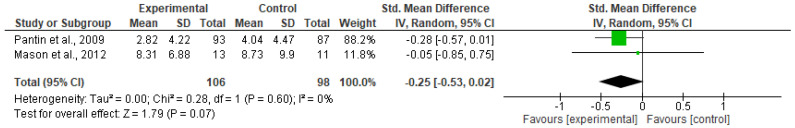
Young people aged 12–14: Internalizing symptoms.

**Figure 10:**
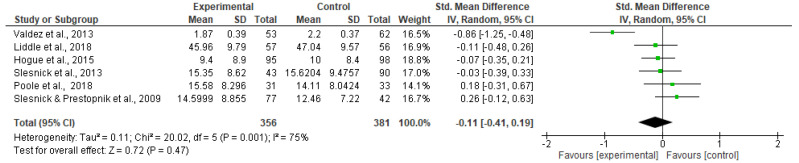
Young people aged 15–17: Internalizing symptoms.

The effects of the intervention upon internalizing symptoms were examined by age of the participants, analyzing separately those interventions aimed at young people aged 12 to 14 and those aimed at young people aged 15 to 17. Results remained non-significant; neither the interventions aimed at the lower age range or the upper age range reduced the internalizing symptoms. Please see Figure 9 and Figure 10.

(2b) Mental Health: Externalizing symptoms (*n* = 11 trials; 2 targeted and 9 treatment)

##### Effectiveness of Family-Involved Interventions

There was no significant difference between the family-involved interventions and the control groups at the longest follow-up time point (SMD −0.26; 95% CI −0.66 to 0.15; *p* = 0.22; 11 trials; 1163 participants)—(see Figure 3). There was considerable and significant heterogeneity (I^2^ = 91% *p* < 0.10).

##### Impact of Prevention and Treatment

**Figure 11:**
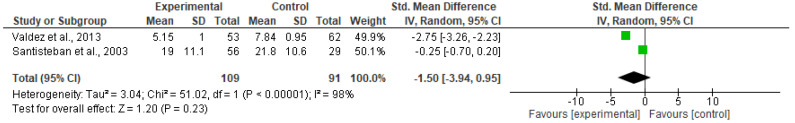
Targeted interventions: Externalizing symptoms.

**Figure 12:**
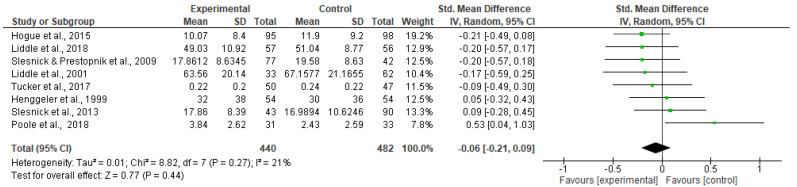
Treatment: Externalizing symptoms.

The effects of the intervention upon externalizing symptoms were examined by level of prevention and treatment, analyzing separately targeted interventions and treatment-based interventions. Results remained non-significant; neither targeted nor treatment-based family-involved interventions reduced externalizing symptoms. Please see Figure 11 and Figure 12.

#### 3.3.2. Secondary Outcomes

(3) Family-conflict (*n* = 6; 2 targeted and 4 treatment)

##### Effectiveness of Family-Involved Interventions

Family-involved interventions reduced family conflict with a small effect compared to control groups (SMD −0.30; 95% CI −0.51 to −0.09; *p* = 0.005; 6 trials; 552 participants)—(see Figure 4) with low heterogeneity (I^2^ = 27% *p* = 0.23).

##### Impact of Prevention and Treatment

**Figure 13:**
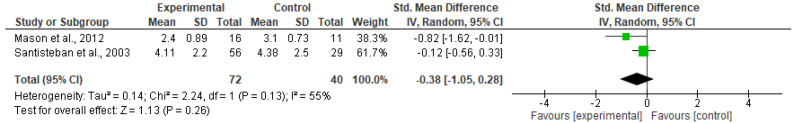
Targeted prevention: Family conflict.

**Figure 14:**
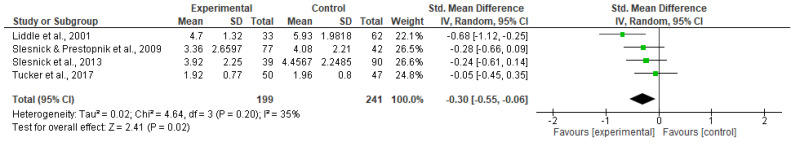
Treatment: Family conflict.

The effects of the intervention on family conflict were examined by level of prevention and treatment, analyzing separately targeted interventions and treatment-based interventions. Results showed that treatment-based interventions were associated with reduced levels of family conflict (SMD −0.30; 95% CI −0.51 to −0.09; *p* = 0.02;4 trials; 440 participants), with no significant heterogeneity (I^2^ = 35% *p* = 0.20). Targeted interventions did not significantly reduce family conflict. Please see Figure 13 and Figure 14.

(4) Frequency of substance use in the past 30 days (*n* = 9 trials; 3 targeted and 6 treatment)

##### Effectiveness of Family-Involved Interventions

There was no significant difference between the family-involved interventions and the control groups at the longest follow-up time point (SMD −0.33; 95% CI −0.72 to 0.06; *p* = 0.10; 8 trials; 761 participants—(see Figure 4). There was considerable and significant heterogeneity (I^2^ = 88% *p* < 0.10).

##### Impact of Prevention and Treatment

**Figure 15:**
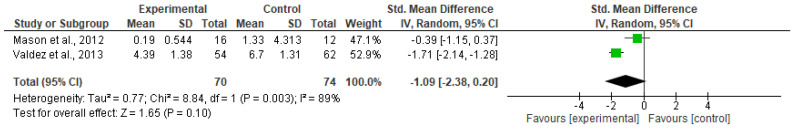
Targeted: Substance use in the past 30 days.

**Figure 16:**
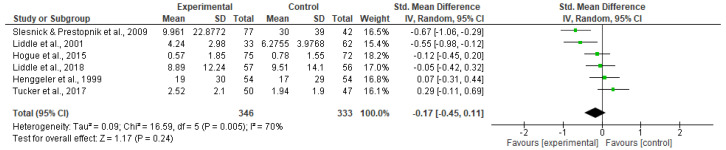
Treatment: Substance use in the past 30 days.

The effects of the intervention on the frequency of substance use were examined by level of prevention and treatment, analyzing targeted interventions and treatment-based interventions separately. Results remained non-significant; neither targeted nor treatment-based family-involved interventions reduced the frequency of substance use. Please see Figure 15 and Figure 16.

#### 3.3.3. Sensitivity Analysis

##### Time Band of 3–12 Month Follow-Up

Omitting trials that did not fall within a time band of 3–12 months did not change the results for any of the primary or secondary outcomes, apart from increased levels of heterogeneity for all outcomes. Results of all sensitivity analyses can be requested from the authors.

##### Outcome Measures 

Omitting trials not reporting an overall internalizing or externalizing symptom score (most frequently used measure) and trials not reporting on composite measures of substance (most frequently used measure) did not impact the findings. However, heterogeneity was no longer significant for internalizing symptoms (I^2^ = 19%, *p* = 0.30) or externalizing symptoms (I^2^ = 0%, *p* = 0.67). Similarly, omitting trials reporting on overall family functioning rather than family conflict specifically did not have a significant effect. There were, however, slightly reduced levels of heterogeneity. 

##### Outliers 

Outliers were identified through visual inspection of the forest plots. Omitting outliers did not have a significant impact on any of the remaining primary or secondary outcomes. However, heterogeneity was no longer significant for internalizing symptoms (I^2^ = 0%, *p* = 0.44) or externalizing symptoms (I^2^ = 15%, *p* = 0.31). 

#### 3.3.4. Publication Bias

Publication bias was not investigated for any of the meta-analyses due to insufficient numbers of trials in each.

## 4. Discussion

This systematic review and meta-analysis found that family-involved interventions were not significantly more effective than controls in reducing the primary outcomes: frequency of alcohol use internalizing and externalizing symptoms in young people aged 12–17. Nor were they effective in reducing the secondary outcome: frequency of substance use. In contrast, the meta-analysis found that family-involved interventions were significantly more effective than controls in reducing the secondary outcome, family conflict. Thus, demonstrating that interventions were effective in reducing the key mechanism of change, as they set out to. However, despite family-involved interventions significantly reducing family conflict, this, in turn, did not reduce co-occurring alcohol use and mental health problems. This finding should be interpreted with caution as it only remained effective for treatment and not targeted interventions.

The meta-analysis found that primarily addressing family functioning may not be sufficient in preventing/reducing co-occurring alcohol use and mental health problems. This is in line with other recent reviews for alcohol use [66] and antisocial behavior [67]. This finding may highlight the importance of including content targeting young people’s needs directly. The inclusion of such youth-focused components has been found to increase the effectiveness of family-involved interventions in preventing substance use [30]. The interventions included in this review contained limited content targeting young people’s needs. Further, the content varied, including individual functioning and targeting external factors such as peers, education and racial and cultural issues. Consequently, the need for youth-focused components should be explored specifically in relation to the needs of young people with co-occurring alcohol use and mental health problems.

This finding also indicates the need to understand how best to involve family members in interventions targeting co-occurring alcohol use and mental health problems. The deficit/problem maintenance [68] has previously informed the way in which families are involved in interventions. This model emphasizes how carers and family members are considered part of the problem; as such, family dysfunction is targeted [68]. Increasingly, family members are recognized as a substantial resource for the young person [69]. For example, the value of galvanizing familial support has been raised as an important protective factor for preventative mental health interventions to target [70]. As such, family members could be considered to be part of the solution [71]. How to best involve family members could be explored through co-production, a method that has been found important in contributing to the effectiveness of interventions [72].

Further, most interventions in this review mainly involved mothers. Few interventions involved more than one parent participation or family members beyond significant caregivers such as siblings, grandparents, aunties or uncles. This could be a reflection of implicit and explicit gender bias, in which mothers can be assumed to be the primary caregivers [73]. However, increasingly, the importance of alloparents, who are family members other than biological caregivers/primary caregivers within the intervention context, is recognized [74]. Consequently, it is important to not only understand how to best involve family members but also which family members to involve in an intervention. Further, few studies considered the impact of a carer’s own mental health or substance use in relation to young people-related outcomes and intervention effects despite strong evidence of such an impact [75,76]. This also requires further exploration.

Few interventions targeted the link between alcohol use and mental health problems. The relationship between alcohol use and mental health problems is complex and multidirectional. Beyond the common factor model, the sequential model suggests that alcohol increases the risk of mental health problems and vice versa [24]. Further, the bidirectional model outlines that alcohol use and mental health problems impact each other [22]. Therefore, multiple mechanisms may be present, and there is a need for the development of a holistic intervention targeting these multiple mechanisms at play. Additionally, there is a need for interventions to target internalizing problems alongside alcohol use. Very few interventions were performed within the review, despite it being well established that internalizing problems also frequently co-occur with alcohol use [20,77,78].

There were several limitations of the included trials, which impacted the systematic review and meta-analysis. Due to a limited number of trials, preventative interventions and treatment were pooled together. This contributed to high levels of heterogeneity; however, this was addressed through sub-group analysis. Further, the consistent outlier Valdez [54] also contributed to heterogeneity. This may be explained by the included vulnerable population group ‘gang affiliated young people’. As such, it is important to interpret the results with caution. Whilst previous research has found family-involved interventions to be effective, more recent reviews have found similar results to this current review [66,67]. As such, findings are inconclusive, and further research is required. Participants were rarely screened for the co-occurrence of alcohol use and mental health problems. Rather, they are often simply screened for alcohol use or mental health problems. As for outcomes at follow-up, either alcohol use or mental health was measured as a primary outcome, with the other simply measured as a secondary outcome. Therefore, there is a need for consistent use of specific screening and outcome measures for the co-occurrence of alcohol use and mental health problems [79]. In addition, only one intervention was developed outside of the USA. Due to considerable social and cultural differences compared to Europe, there is a need for future interventions to be developed and evaluated outside of the USA [80].

Varying degrees of bias were present across the included trials. Comparators often consisted of active controls, limiting the ability of trials to identify significant intervention effects. Many of those that employed inactive controls included treatment as usual, which often involved alternate therapy. A number of trials, including several pilot feasibility trials, utilized small sample sizes, resulting in the likelihood of underpowered trials and an increased risk of type II error. Three trials included medication administration in addition to family-involved interventions, and one trial provided additional Motivational Enhancement Therapy/Cognitive Behavior Therapy. This limits the ability to attribute effects to the family-involved interventions. However, all control groups were offered the same additional treatment. 

## 5. Conclusions

Family-involved interventions were not effective, compared to controls, in reducing co-occurring mental health and alcohol use problems. This may be due to primarily addressing family functioning as a preceding mechanism, theorized to reduce co-occurring alcohol and mental health problems. Rather, interventions may need to focus on directly targeting young people’s alcohol use, mental health and the link between the two. The findings are inconclusive, and further research is required. Specifically, there is a need for future development and evaluation of country-specific interventions, which should involve co-development with young people, family members and stakeholders. This will also enable the exploration as to how best to involve family members and which family members to involve.

## Figures and Tables

**Table 1:** Characteristics of included trials.

Identifier	Recruitment	Sample	Sample Size	Interventions	Intervention Hours	Control	Outcomes	Follow-Up Time Points	Study Design
**Targeted Interventions**									
**Mason et al., 2012 [46]** **USA**	Healthcare clinics and therapeutic centres	Caregivers screened for elevated depressive symptoms. Mean age: 13.9 years Female: 43.5%	24 adolescents and their families	Project Hope Content: family functioning, parenting techniques and young people’s coping skills/substance refusal skills. Delivery: Separate adolescent and whole family sessions.	10 × 90- min sessions	Waitlist control group	Frequency of alcohol use from Project Family Depressive symptoms: Moods and feelings questionnaire Family conflict: three item scale from Project Family Frequency of marijuana use from Project Family	Post-test	Pilot feasibility Randomised controlled trials—2 arms
**Pantin et al., 2009 [51]** **USA**	Hispanic middle schools	Screened for elevated symptoms using Revised Behaviour Problem Checklist Mean age: 13.8 years Female: 64%	213 8th grade Hispanic adolescents and their families	Familias Unidas Content: Family functioning and parenting techniques Delivery: Adolescent involvement limited to family visits	9 × 2 h group parent sessions, 10 × 1 h family visits and 4 × 1 h booster sessions	Community control: Referrals to agencies providing services behaviour problems.	Composite measure: Frequency of alcohol, cigarette, and marijuana use from monitoring the future study Externalising symptoms: Diagnostic Interview Schedule for Children (DISC) Anxiety-withdrawal subscale of the Revised Behaviour Problem Checklist	6,18 & 30 months post-baseline	Randomised controlled trial—2 arms
**Santisteban et al., 2003 [64]** **USA**	Self-referred or referred by a school counsellor	Complaints of externalising behaviour problems Mean age: 15.6 years Female: 25%	126 Hispanic adolescents and families with	Brief Strategic Family Therapy Content: Family functioning Delivery: Whole family sessions	Approximately 20 × 1 h weekly sessions (amount dependent on the clinical severity)	The Group Control Condition: Participatory learning group. 6–16 sessions × 90 min.	Frequency of alcohol use: Addiction Severity Scale Externalising symptoms: Revised Behaviour Problem Checklist The Conflict Scale: Family Environment scale Frequency of marijuana use: Addiction Severity Scale	Post-test	Randomised trial—2 arms
**Valdez et al., 2013 [54]** **USA**	Field-intensive outreach and street-based recruitment	Gang-affiliated with current use of alcohol or illicit drugs Intervention: Mean age: 15.33 years Female: 31.7% Control: Mean age: 15.18 years Female: 51%	200 Mexican-American adolescents and their families.	Adapted Brief Strategic Family Therapy Content: Family functioning, school engagement, gang diversion and awareness. Delivery: Family, caregiver and adolescent sessions.	12–16 × 1–1.5-h sessions	Social and Behavioural Health Services and Substance Abuse Counselling: Some family involvement.	Frequency of alcohol use: Centre for Substance Abuse Treatment’s Government Performance and Results Act-Client Outcome Questionnaire Externalising symptoms: Conners’ Rating Scale Frequency of marijuana use: see above scale used for alcoYeshol use	Post-test & 6 months post-test	Randomised trial—2 arms
**Treatment based interventions**									
**Henggeler Pickrel and Brondino, 1999 [57]** **USA**	Department of Juvenile Justice	Juvenile offenders with substance abuse or dependence Mean age: 15.7 years Female: 21% Minority/multiracial: 53% Caucasian: 47%	118 adolescents and their families	Multisystemic Therapy Content: Family functioning and parenting techniques. Delivery: Family composition within sessions is not provided. Medication available	Length determined by clinical need.	Usual Community Services: Outpatient substance use services and/or mental health inpatient and outpatient services	Composite measure: Frequency of alcohol and marijuana use: Personal experience inventory Delinquency Scale	Post-test and 6 months post-test	Randomised controlled trial
**Hogue et al., 2015 [45]** **USA**	Community referral network	Adolescents who met criteria for either mental health or substance use problems. Mean age: 15.7 years Female: 48% Minority/multiracial: 80%	205 adolescents and their families.	Non-manualised Family Therapy Content: Family functioning and parenting techniques Delivery: Whole family sessions only.	Average of 9 sessions	Usual Care Other: Access to five outpatient clinics. Weekly treatment sessions and psychiatric support.	Composite measure: Frequency of alcohol or illegal drugs: Timeline Follow Back Method Internalizing symptoms: Child Behaviour Checklist Externalising symptoms: Child Behaviour Checklist	3,6,9,12-months post-baseline	Randomised controlled trial—2 arms
**Liddle, 2001** **USA [59]**	Juvenile justice systems, schools, health and mental health agencies	Adolescents who were using any illegal substance other than alcohol at least three times per week. Alcohol use could be greater or less than three times per week. Mean age: 15.9 years Female: 20% Caucasian: 51% Minority/multiracial: 49%	182 adolescents and their families.	Multidimensional Family Therapy Content: Family functioning and parenting techniques Delivery: Family, caregiver, and adolescent sessions.	14–16 weekly 90-min sessions	Adolescent Group Therapy: 14–16 weekly 90 min sessions. Included family sessions. Multifamily educational intervention 14–16 weekly 90 min group family sessions. Individual family crisis sessions	Composite measure: Frequency of alcohol and marijuana use-Timeline Follow Back Method Acting out behaviour scale Family Conflict: Degree of health and dysfunction of behavioural family transactions, Global Health Pathology Scale of the Beavers Interactional Competence Scales	12 months post-intervention	Randomised controlled trial
**Liddle et al., 2018 [58]** **USA**	Referred and approved by Department of Children and Families	Adolescents diagnosed with a substance use disorder and a comorbid psychiatric disorder warranting a higher level of care. Mean age:15.36 years Female: 25% Minority/multiracial: 86% Caucasian: 13%	113 adolescents and their families.	Multidimensional Family Therapy Content: Family functioning and parenting techniques. Psychiatric care and medication management also available. Delivery: Family, caregiver and adolescent sessions.	3.28 h of sessions per week	Residential Substance Use Treatment: Included monthly parental support groups. Psychiatric care and medication available	Composite measure: Frequency of any drug use including alcohol: Timeline Follow Back Method Internalizing symptoms: Child behaviour checklist Externalising symptoms: Child behaviour checklist ∙	2,4,12,18-month post-baseline	Randomised controlled trial
**Poole et al., 2018 [47]** **USA**	Public mental health service, schools, and community mental health service	Young people with depression Mean age: 15.2 years Female: 73.4% Ethnicity not reported	64 adolescents and their families	Best Mood-Behavior Exchange Systems Therapy for adolescent depression Content: Family functioning, parenting techniques and young people’s coping skills Delivery: Half of the sessions involved adolescents and siblings.	8 × 2 h multifamily group sessions and 1 × 2 h follow-up session	PAST (treatment as usual): Aimed to represent treatment as usual in Victoria, Australia, parenting groups	Problematic alcohol drinking behaviour: Alcohol Use Disorders Identification Test Conduct problem subscale: The Strengths and Difficulties Questionnaire The Short Moods and Feelings Questionnaire	3 months post- treatment	Randomised trial—2 arms
**Slesnick and Prestopnik., 2009 [48]** **USA**	Runaway Shelters	Adolescents with primary alcohol problems (‘for example, alcohol dependence and marijuana abuse but not vice versa’). Mean age: 15.1 years Female: 55% Minority/multiracial: 83%	119 adolescents and their families	Ecologically-Based Family Therapy (EBFT) Content: Family functioning and parenting techniques Delivery: Home-based, Family, caregiver and adolescent sessions. Functional Family Therapy Content: Family functioning and parenting techniques. Delivery: Office-based. Whole family sessions only.	16 × 50 minute sessions	Service As Usual: Mainly case management and informal meetings or therapy provided/arranged by shelter staff.	Frequency of alcohol use: The form 90 Internalizing symptoms: Child Behaviour Checklist Externalising symptoms: Child Behaviour Checklist Composite measure: Frequency of alcohol and drug use: The form 90 Family conflict: The family Environment scale	3,9,15 months post-baseline	Randomised controlled trial—3 arms
**Slesnick** **et al., 2013 [49]** **USA**	Runaway shelter	Met criteria for alcohol abuse or dependence Mean age: 15.4 years Female: 52.5% African American: 65.9%, Caucasian: 26%	179 young people and their families	Ecologically-Based Family Therapy (EBFT) Content: Family functioning and parenting techniques. Delivery: Family, caregiver and adolescent sessions.	14 sessions	Community Reinforcement Approach (skills training): 14 sessions Motivational Interviewing: 2 sessions	Composite measure: Frequency of alcohol and drug use: The form 90 Internalizing symptoms: Child behaviour checklist Externalising symptoms: Child behaviour checklist	3,6,9, 12,18,24 months post-baseline	Randomised trial—2 arms
**Stanger** **et al., 2017 [55]** **USA**	Schools, justice system, therapists, physicians, or parents.	Met criteria for alcohol abuse or dependence Mean age:16.1 years Female: 25.35% Caucasian: 81%	75 young people and their families	Abstinence-based Fishbowl Program, Home-based Incentives and Consequences Program Content: Parenting techniques, incentives, and consequences for young people’s substance use. All young people also received individual MET/CBT Delivery: Separate sessions with caregivers and young people only.	Number of sessions was not reported	Attendance Based Incentives: Number and duration not provided. All young people received additional individual MET/CBT	Composite measure: Frequency of alcohol and marijuana use- Time Line Follow Back Method Externalizing symptoms: Child Behaviour Checklist	36 week follow up	Randomised controlled trial—2 arms
**Tucker** **et al., 2016 [56]** **USA**	A community-based agency	Adolescents with ‘poor grades, truancy, defiant behaviour, delinquency or substance use’. Mean age: 14.97 years Female: 44.89% Minority/multiracial: Hispanic: 99% Caucasian: 1%	111 young people and their families	Parent-Child Mediation Content: Family functioning. Delivery: Family, caregiver, and adolescent sessions.	3 mediation sessions	Waitlist Control Group	Frequency of alcohol use Externalising symptoms: items from Project Alert Frequency of marijuana Family Conflict Scale	1.5, 3 months post-baseline	Pilot feasibility randomised controlled trial—2 arms
**Wolff et al., 2020 [61]** **USA**	A community mental health clinic	Adolescents with co-occurring substance use and mental health problems Mean age: not provided Female: 42% Minority/multiracial: 31%	111 young people and their families	Integrated-Cognitive Behavioural Treatment Content: Family functioning, parenting techniques, coping skills and alcohol refusal skills in young people. Delivery: Family, caregiver, and adolescent sessions.	30 min-3 h, up to 3 times a week.	Treatment as Usual: Could include eclectic, flexible treatment	Frequency of alcohol use: Adolescent Drinking Questionnaire Depression symptoms: Child depression inventory-2 Externalising symptoms: The child behaviour checklist	3,6,12 months post-baseline	Randomised controlled trial—2 arms

## Data Availability

Data available upon reasonable request.

## Supplementary Materials

The following supporting information can be downloaded at: https://www.mdpi.com/article/10.3390/ijerph20196890/s1, File S1: search strategy.
