# Supplementary material for: Effectiveness of Family-Involved Interventions in Reducing Co-Occurring Alcohol Use and Mental Health Problems in Young People Aged 12–17: A Systematic Review and Meta-Analysis
Source: Int J Environ Res Public Health. 2023 Oct 6;20(19):6890. doi: 10.3390/ijerph20196890 (PMC10572317; doi:10.3390/ijerph20196890)
Supplement: Supplementary file 1 [file ijerph-20-06890-s001.zip › ijerph-2610360-supplementary.pdf]

1. *adolescent/
2. *alcohol drinking/ or *binge drinking/ or *underage drinking/ or *alcohol-Related Disorders/
3. *mental Health/ or *"mental disorders"/ or *anxiety disorders/ or *"disruptive, impulse control, and conduct disorders"/ or *mood disorders/ or *"attention deficit and disruptive disorder with hyperactivity"/ or *conduct disorder/ or *aggression/ or *depression/ or *stress, psychological/
4. ((mental or psychologic* or behavio?ral or emotion*) adj2 (ill* or disorder* or symptom* or health or problem* or difficult*)).ab,ti.
5. 2 or 3 or 4
6. 1 and 5
7. ((teen* or pre?teen or adolescen* or youth or "young person" or "young people" or young* or child* or juvenile* or underage*) adj5 (alcohol* adj2 (drink* or intoxicat* or use* or abus* or misus* or risk* or consum*))).ab,ti.
8. ((teen* or "pre-teen" or adolescen* or youth or "young person" or "young people" or young* or child* or juvenile* or underage*) adj5 (drink adj2 (binge or harmful or problem))).ab,ti.
9. ((teen* or "pre?teen" or adolescen* or youth or "young person" or "young people" or young* or child* or juvenile*) adj5 (anxiet* or anxious* or stress or stressed or depress* or self?harm* or "self?injur*" or suicid* or resilience or self?esteem or internali?ing or externali?ing or impuls* or "conduct disorder" or "oppositional defiant disorder" or "attention deficit hyperactivity disorder" or "attention?deficit disorder" or anger* or aggress* or "anti?social behavio?r" or "family functioning" or "family relationship" or "problem behavio?r" or well?being or "quality of life")).ab,ti.
10. 7 or 8 or 9
11. family/ or parents/
12. (famil* or parent or parents or relative or relatives or sibling* or grandparent* or family?centered or family? centred or family?based or family?involved or family?strengthening).ab,ti.
13. 11 or 12
14. family therapy/
15. (reduc* or increas* treatment* or therap* or preven* or promot* or screen* or psychoeducat* or psychosocial or intervention* or program* or counsel* or (systemic adj2 therap*) or (multi?systemic adj2 therap*) or "behavio?ral intervention*" or "behavio?ral therap*").ab,ti.
16. 14 or 15
17. 6 or 10
18. 13 and 16 and 17
19. randomized controlled trial.pt.
20. controlled clinical trial.pt.
21. randomized.ab.
22. placebo.ab.
23. clinical trials as topic.sh.
24. randomly.ab.
25. trial.ti.
26. 19 or 20 or 21 or 22 or 23 or 24 or 25
27. exp animals/ not humans.sh.
28. 26 not 27
29. 18 and 28
